# Solar fields in farmlands, their impact on bat presence and activity

**DOI:** 10.1371/journal.pone.0335581

**Published:** 2026-06-01

**Authors:** Chloé Tavernier, Rascha Nuijten, Ralph Buij, Frank van Langevelde

**Affiliations:** 1 Wildlife Ecology and Conservation, Wageningen University and Research, Wageningen, The Netherlands; 2 Future For Nature Foundation, Antoon van Hooffplein 1, Arnhem, the Netherlands; 3 Animal Ecology, Wageningen University and Research, Wageningen, The Netherlands; University of Udine: Universita degli Studi di Udine, ITALY

## Abstract

Bat populations are facing numerous challenges due to human activities, and the development of solar energy in agricultural landscapes may add to these issues. Effects of solar fields on bat populations are still poorly understood. In this study, we compared bat activity in six solar fields that each had two grassland controls (agricultural grassland and natural meadows). Using passive bat detectors, bat calls were recorded along the edges of these plots, where the highest bat species diversity and activity were expected. The effects of landscape composition and configuration on the diversity, presence, and activity of different bat species were evaluated for each plot type at yearly, seasonal, and monthly scales. All seven bat species studied were less active in the solar fields compared to the two grassland controls. Specifically, both the number of nights that bats were present and the activity of bats were reduced in solar fields. As the number of solar fields keeps increasing in the agricultural landscape, it is thus essential to monitor their effect on the population levels.

## Introduction

Globally, bats face numerous threats from human activities, with agriculture and energy production ranking respectively as the second and fourth most significant contributors to their decline [1]. In particular, insectivorous bat species are negatively affected by intensive agricultural practices, as studies have shown that their foraging activity is lower on conventionally managed farmland compared to organic farmland, likely due to insecticide use [2]. In addition to habitat loss, bats are directly impacted by various human activities. For example, in Europe, bat mortality caused by wind turbines is well-documented [3,4]. Beyond the effects of wind turbines on local bat populations, there is growing concern that fatalities among migratory bat species could elevate their risk of extinction [1]. Bat species are under legal protection in many countries [5,6].

Besides the use of wind turbines, solar energy capacity is growing worldwide [7]. However, the impact of solar fields (i.e., structures of photovoltaic panels arranged in rows) on biodiversity remains largely unexplored [8,9]. Their primary impact on biodiversity is expected to mainly result from habitat modification [10,11]. In the Netherlands, for example, where 52% of the land is used for agriculture, solar fields are predominantly established within conventional agricultural landscapes [12], and this trend is the same at the European scale [13]. Some studies suggest that solar fields have mainly negative effects on bat populations. In particular, bats exhibit significantly lower foraging activity within solar fields compared to nearby control sites [14]. Moreover, it has also been found that other types of solar energy production, like concentrated flux, can contribute to bat mortality [15]. It is also a risk discussed for solar fields due to the potential lake effect created by the solar panel that could attract water insects and drinking bats. So far, research comparing bat activity in solar fields to local controls has, however, yielded mixed results. In southwest England, for example, most bat species showed reduced activity in solar fields compared to grassland controls, both within the fields and along adjacent hedgerows [16]. In contrast, a study in Hungary found that most bats, with the exception of *Myotis* spp., exhibited similar activity levels in solar fields and grassland controls [17]. The positive or negative effect of solar fields on bats likely reflects species-specific traits, such as variation in foraging habitats, susceptibility to predation, and flight capabilities, as well as local agriculture systems.

Nevertheless, converting intensively farmed land into a solar field could offer potential benefits for bats, as found for other mammal species (Tavernier et al., forthcoming). Indeed, unlike agricultural production, pesticides or herbicides are often not applied in solar fields, which could promote local insect diversity ([18], personal communication CT with solar park developers). Additionally, most solar fields are enclosed by hedgerows to minimise visual impact in the surrounding landscape ([19], S1 Table in S1 File). Hedgerows, often absent in intensively farmed areas, play a crucial role in bat dispersal and habitat connectivity [20,21]. The integration of solar fields into homogeneous agricultural landscapes could, therefore, contribute to the restoration of bat species’ habitat.

Two studies on the effects of solar fields on bat have been performed by Tinsley et al. [16] and Szabadi et al. [17]. However, both studies were conducted during the summer months (July to October and July to September, respectively). Notably, this period coincides with peak bat activity in agricultural landscapes, as bats have been shown to increase their use of farmland after July [22]. However, spring is also a critical season, as bats shift their habitat selection during lactation [23]. It is, therefore, possible that solar fields are used at varying intensities across seasons, potentially serving as alternative habitats when agricultural activity fluctuates in surrounding fields. Thus, this study aimed to assess the potential effects of solar fields on bat diversity, yearly bat occurrence, and seasonal (spring, summer, autumn) and monthly bat activity. In particular, as bat species may respond differently to the presence of solar fields due to their distinct ecology, the study examines the impact at the species level. To evaluate this impact, occurrence and activity in solar fields were compared with those in two control fields, an intensively managed grassland and a protected meadow. As solar fields are located in farmlands, the two control types were selected as they might represent a possible alternative to land use from solar fields in the Netherlands.

## Method

### Sampling design

**Fig 1:**
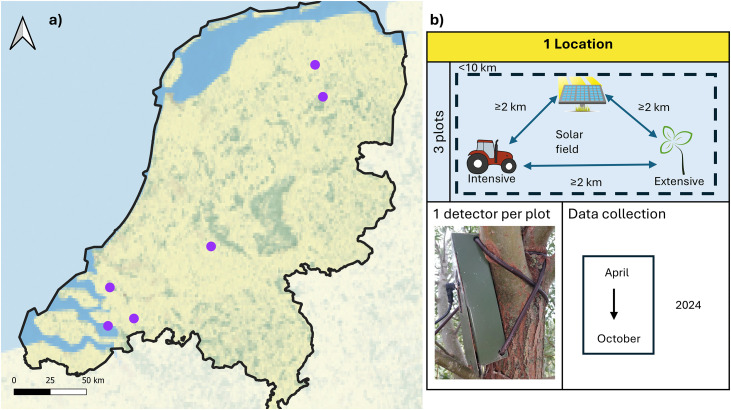
Sample design in seven solar fields and their controls. **(a)** Satellite image of the Netherlands from Natural Earth (https://www.naturalearthdata.com/) and the six locations where the bat survey took place. **(b)** Description of the survey design, where each location was composed of three plots: the solar field and the two control fields, being an extensive meadow and an intensive grassland. The Clip art are from https://openclipart.org/.

A block-plot design was implemented at six locations to evaluate the impact of solar fields on bat species diversity, presence and activity in the Netherlands. The solar fields were managed by different developers, resulting in variations in vegetation management and panel layouts. Access was restricted to the public in all solar fields, and they were all surrounded by hedgerows in various stages of development (S1 Table in S1 File). Solar field developers provided authorisation and access for the research. In each location, three plots were surveyed: a solar field and two grassland controls (Fig 1). The two controls were an intensively managed grassland for dairy production (intensive control) and an extensively managed meadow for nature conservation (extensive control). Authorisation was provided by farmers or field managers before the survey. The controls were located between two and ten kilometres from the solar fields. These distances were to reduce the effect of solar fields on the bat presence in the controls while staying in roughly the same landscape. Each plot was located in the agricultural landscape, meaning that the matrix surrounding each plot was dominated by farmland. Authorisation to place the detector and visit each plot was granted by their owners.

### Bat survey

In each plot, a stand-alone bat detector (Anabat Swift, www.titley-scientific.com) equipped with an omnidirectional microphone (US-O V3 [10–140 kHz], www.titley-scientific.com) was placed from the 1^st^ April to the 2^nd^ December in 2024 (S1 Fig). The detectors were set up to automatically start recording full-spectrum data from 30 mins before sunset until 30 mins after sunrise. The batteries and SD cards were changed once a month. The detectors were placed on poles or trees at the edge of the plots at a height of two meters above the ground. They were not calibrated to each other.

Sound files were analysed in two steps. First, automated identification was performed by Kaleidoscope Pro [24] using the European classifier with a conservative threshold. All *Myotis* and *Plecotus* species were also classified under *Myotis* spp. and *Plecotus* spp. respectively, as they are known to be difficult to discriminate based on their echolocation [25]. Second, one percent of each species’ sound files were then manually annotated to estimate the error produced by the automatic classifier (S2 Table in S1 File). Then species or noise that could not be identified by the classifier were manually processed using Anabat Insight [26]. However, only *Pipistrellus nathusii*, *Pipistrellus pipistrellus*, *Pipistrellus pygmaeus*, *Nyctalus noctula*, and *Eptesicus serotinus* species, as well as *Myotis spp.* and *Plecotus spp.,* were considered in this last classification phase (Table 1) as they are the species the most reliably determined through sound recording. Bat calls were then resampled into events. An event was defined as a cluster of calls from the same species less than three seconds apart [27].

**Table 1:** Species included in the study and their corresponding preferred foraging habitat, as well as their migratory behaviour. Classification followed Barré et al. (2024) and Heim et al. (2016) classifications. Occurrence refers to the number of nights a species was detected. Ratio Activity refers to the total event from one species divided by the total number of events (165724).

Species	Foraging habitat	Migratory behaviour	Occurrence	Ratio Activity
*Eptesicus serotinus*	Open	Sedentary	551	0.026
*Myotis spp.*	Narrow	Regional or Sedentary	721	0.013
*Nyctalus noctula*	Open	Migratory	884	0.077
*Pipistrellus nathusii*	Edge	Migratory	11177	0.097
*Pipistrellus pipistrellus*	Edge	Sedentary	12777	0.780
*Pipistrellus pygmaeus*	Edge	Migratory or Sedentary	121	0.001
*Plecotus spp.*	Narrow	Sedentary	419	0.006

### Landscape and land use data

The area of different land uses essential for bats in three buffer zones was measured to test the effect of the surrounding landscape composition and configuration on bat communities in each of the plots [28,29]. The three buffers were set at 500 m, one km and two km from each plot based on the minimum distance between two plots and the effects observed in other studies [20,21]. We did not test the effect of the landscape further than two km away, as this was the minimum distance between the solar field and the two grassland controls, meaning that beyond this distance, we could not distinguish the separate effects of surrounding land uses on the plots anymore. Land uses considered in the landscape were “Forest”, “Shrubs” and “Fresh water” as they are particularly relevant for bat movement and foraging activities. They were extracted and reclassified from the land use map of the Netherlands, LGN2023 (Hazeu et al., 2023). It was found that areas covered by tall vegetation and connectivity were particularly relevant to explain bat abundance in farmlands [30]. Thus, the landscape composition was defined by the amount of habitat present in the buffer zone, and configuration by the distance between two patches of the same habitat, as well as the minimum distance between each plot and the habitats. The metrics were calculated using the *landscapemetric* package in R [31] or QGIS [32].

### Statistical analysis

All the analyses were conducted using R Statistical Software [33]. The analyses were conducted on each species or genus (Table 1).

The effect of landscape composition and configuration on species composition was investigated using a redundancy analysis (RDA). The RDA was performed to test the influence of environmental covariates on species composition in the plots. Environmental metrics, as well as the type of pole the detector was attached to (Table 2), were tested for correlation using a 0.8 threshold and collinearity using a variance inflation factor (VIF) below ten. Step selection was performed on the full and empty model using the *ordistep* function from the *vegan* [34] package, with a limit of permutation of P-values < 0.1 for a term to be added in the final model. A generalised linear model (GLM) using the *glmmTMB* package [35] was used to test the effect of selected landscape metrics on each bat species activity (log-transformed), with the plot set as a random factor and a temporal covariate to account for seasonal change.

**Table 2:** Landscape metrics from the *landscapemetric* package in R [31] and land-use definition used in the present study.

Name	Units	Description
*Total area (ca)*	Hectare	Sums the area of all patches belonging to class i.
*Mean of euclidean nearest-neighbour distance (enn_mn)*	Meter	Mean of ENN. ENN measures the distance to the nearest neighbouring patch of the same class i. The distance is measured from edge to edge. The range is limited by the cell resolution on the lower limit and the landscape extent on the upper limit.
*Minimum distance (dist)* ^*a*^	Meter	Shorter distance between the plot and the nearest neighbouring patch of class i.
*Port*		Define whether the detector was set on a tree or not.
***Land-use classes***	***Description***	
*Forest*	Patches dominated by trees	
*Fresh Water*	Waterways, rivers, and lakes	
*Shrubs*	Patches of small trees or hedgerows	

^a^The minimum distances were manually measured using GIS software.

Bat diversity in each plot was measured by Hill numbers for abundance data (equation 1) [36]. Three Hill numbers were calculated for each plot with q of order 0 (species richness), 1 (Shannon index), and 2 (Simpson index) using the *iNext* package [37].


$$\begin{array}{c} qD={(\sum \overset{S}{\underset{i=\text{1}}{\mathrm{\backslash nolimits}}}\overset{q}{\underset{i}{p}})}^{\text{1}/(\text{1}-q)} \end{array}$$ (1)

With N, S and p the parameters representing the true assemblage size, the species absolute abundance sets and the relative abundance sets, respectively [36].

The effect of solar field on bat activity (i.e., number of events per night), bat occurrence (i.e., presence during the night), and bat diversity against the controls was assessed using GLMMs. Bat occurrence and activity were measured at two temporal levels, namely yearly and seasonally. GLMMs were fitted with a negative binomial distribution on the activity and with a binomial distribution on the occurrence, except for *P. pipistrellus* activity, which was fitted with a Gaussian distribution. Location, the combination of a solar field and its two controls, was included as a random factor. The yearly efforts or a temporal covariate (week number or Julian day) were incorporated as covariates when the Akaike Information Criterion improved by 2 or more. Indeed, bat detectors were not active the same number of nights (min = 33 nights, max = 153 nights,) and activity can change depending on the season. As *Myothis* spp. and *Plecotus* spp. were rare, a zero-inflated generalised linear mixed model was implemented. The model details are reported in Table 3.

**Table 3:** Formulas of the generalised linear mixed model (GLMM) with a binomial or negative binomial distribution assessing the effect of plot types on species presence or activity, respectively. The models were performed for each species separately. Location (n = 6) was incorporated as a random variable.

Species	Time scale	Response Variable	Covariates
*P.pipistrellus*	Yearly	Presence	~Plot type + (1|Location) + offset(log(Yearly effort)
		Activity (Gaussian)	~Plot type + Yearly effort + (1|Location)
	Seasonal	Presence	~ Plot type * Season + (1|Location) + log(Effort)
		Activity (Gaussian)	~ Plot type * Season + (1|Location) + log(Effort)
*P.nathusii*	Yearly	Presence	~Plot type + (1|Location
		Activity	~ Plot type + Julian day + (1|Location)
	Seasonal	Presence	~ Plot type * Season + (1|Location) + log(Effort)
		Activity	~ Plot type * Season + (1|Location) + log(Effort)
*P.pygmaeus*	Yearly	Presence	~Plot type + (1|Location) + offset(log(Yearly effort)
		Activity	~Plot type + Yearly effort + (1|Location)
	Seasonal	Presence	~ Plot type * Season + (1|Location) + log(Effort)
		Activity	~ Plot type * Season + (1|Location) + log(Effort)
*N.noctula*	Yearly	Presence	~Plot type + (1|Location) + offset(log(Yearly effort)
		Activity	~Plot type + Julian day + (1|Location)
	Seasonal	Presence	~ Plot type * Season + (1|Location) + log(Effort)
		Activity	~ Plot type * Season + (1|Location)
*E.serotinus*	Yearly	Presence	~Plot type + (1|Location)
		Activity	~Plot type + (1|Location)
	Seasonal	Presence	~ Plot type * Season + (1|Location) + log(Effort)
		Activity	~ Plot type * Season + (1|Location) + log(Effort)
*Myotis sp.*	Yearly	Presence	~Plot type + (1|Location)
		Activity	~Plot type + (1|Location)
	Seasonal	Presence (zi=~1)	~ Plot type * Season + (1|Location) + log(Effort)
		Activity (zi=~1)	~ Plot type * Season + (1|Location) + log(Effort)
*Plecotus sp.*	Yearly	Presence	~Plot type + (1|Location) + offset(log(Yearly effort)
		Activity	~Plot type + (1|Location) + offset(log(Yearly effort)
	Seasonal	Presence	~ Plot type * Season + (1|Location) + log(Effort)
		Activity (zi=~1)	~ Plot type * Season + (1|Location) + log(Effort)

## Results

The automatic classifier for bat calls found three species that we did not include in the analyses: *Eptesicus nilssonii*, *Nyctalus leisleri*, and *Vespertilio murinus*. From the included bats (Table 1), a total of 165 724 events were recorded during the 1318 detector nights (i.e., the number of nights a detector was active) of the survey, with each plot being sampled from 33 to 153 nights between April and November (S1 Fig). 78% of the events were records of *P. pipistrellus* (Table 1). The kaleidoscope classifier truly annotated *E.serotinus* at 82%, *N.noctula* at 95%, *P.nathusii* at 88%, *P.pipistrellus* at 99%, *P.pygmaeus* at 63%, *Myotis spp.* at 76%, and *Plecotus spp.* at 80% (S2 Table in S1 File) All the model results are reported in Supporting Information 3–6.

**Fig 2:**
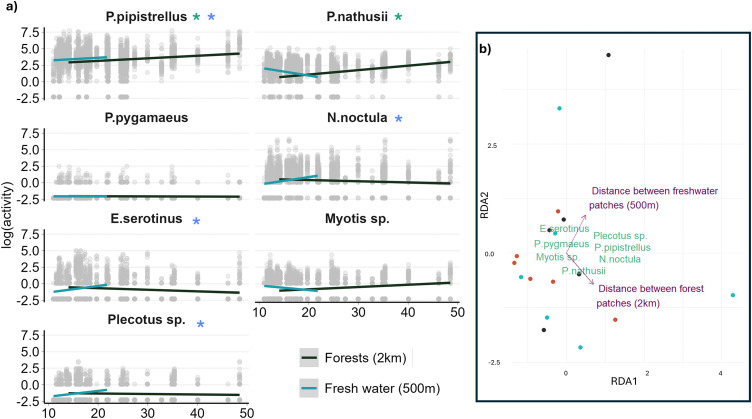
Bats’ activity and the landscape metrics. **a)** Bat activity per night related to the distance between the forest (green) or freshwater patches (blue) in the landscape. Forest patches are within a 2 km buffer, while freshwater patches are within a 500m buffer around each sampling plot. Activity is log-transformed, and distance is expressed in meters. Lines show the linear regression. Significance of the landscape metric on activity is expressed by a star. **b)** RDA output of the species composition in different plots. Species are in green in the following format: Genus.species. The constrained metrics explaining 24% of the variation found in species composition are shown in purple. The landscape metrics are calculated in a buffer around each plot; the size of the buffer is in brackets (500m or 2 km).

When considering occurrence, no landscape metrics were selected using the RDA during the step-selection that would explain community composition. For activity, the two environmental variables selected for the final RDA to explain community composition were: the distance between forest patches within a two-kilometre buffer area and the distance between freshwater patches within a 500m buffer area. The final RDA, composed of the two selected variables, represented 24.0% of the total variation in species activity between plots. The effect of the distance between forest and water patches was species-specific (Fig 2, S3 Table in S1 File).

**Fig 3:**
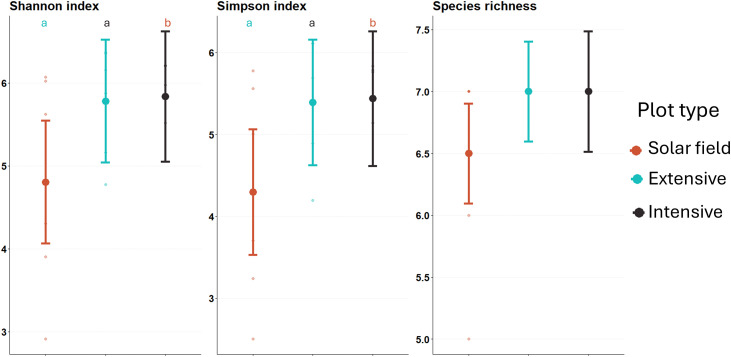
Estimated marginal means of the Hill numbers in the three plot types. Hill numbers with q = 0 (Species richness), q = 1 (Shannon diversity), and q = 2 (Simpson diversity) calculated in solar fields (in red), extensive controls (in blue), and intensive controls (in black). Points represent the estimated Hill numbers from the GLMM, and bars represent the 95% confidence intervals. Note the differences in scale of the y-axis for each species. Significant differences between plot types are indicated by different letters (p < 0.05), with letter order representing the direction of the effect **(a > b)**. No letter indicates no significant difference.

The solar field and the two controls had the same species richness (7 species), meaning that all species visited the three plot types. However, there was strong evidence of a more uneven coverage of species in solar fields due to hyperabundance of *P. pipistrellus*, as indicated by the lower Shannon and Simpson diversity indices compared to the extensive and intensive controls (Fig 3, S4 Table in S1 File).

**Fig 4:**
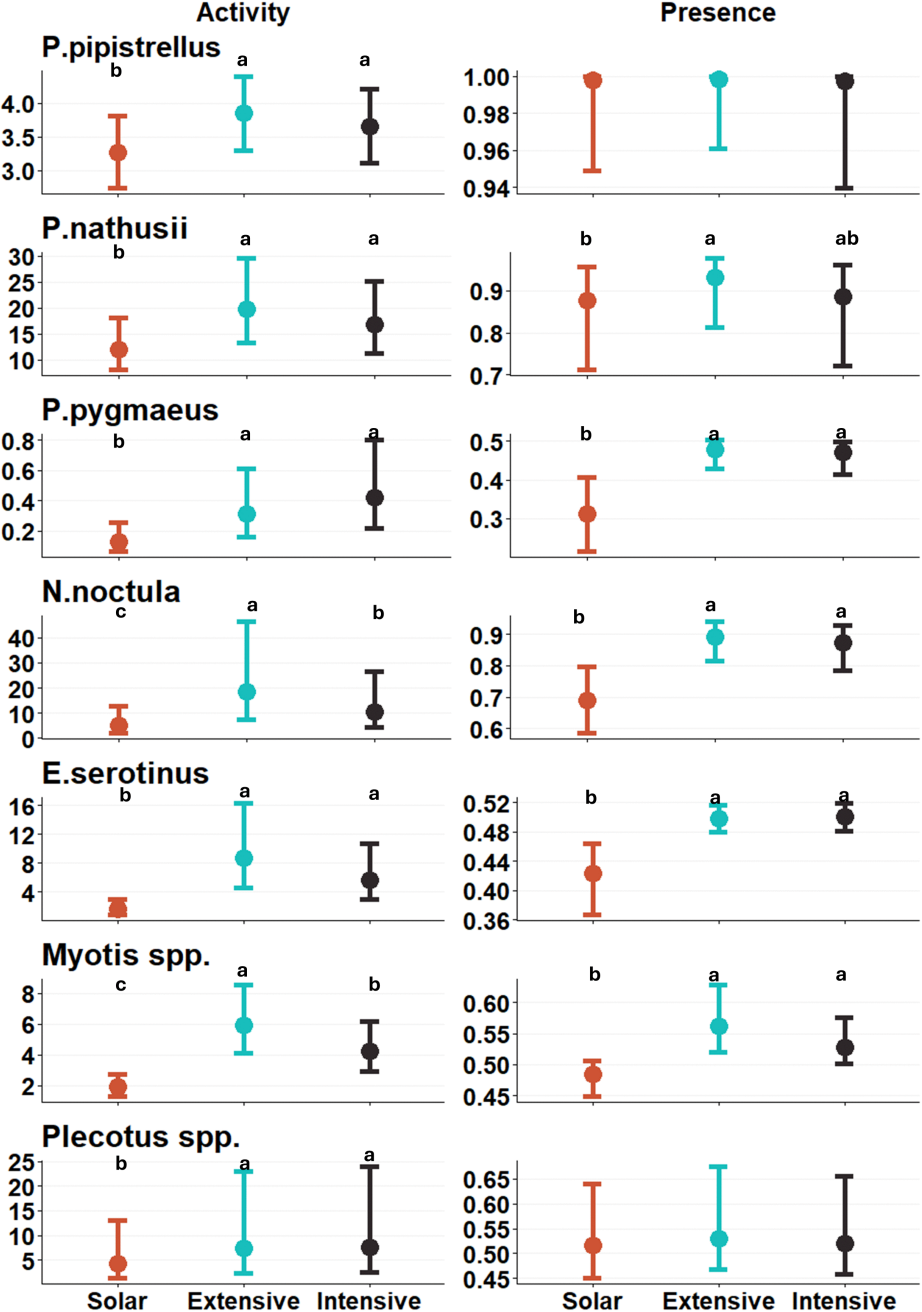
Estimated marginal means of bat activity (left) and occurrence (right) of each species in the three plot types. The plot types are solar fields (in red), extensive controls (in blue), and intensive controls (in black). Points represent the estimated activity and occurrence from the GLMM, and bars represent the 95% confidence intervals. Note the differences in scale of the y-axis for each species. Different letters express P-values less than 0.05, with letter order representing the direction of the effect **(a > b)**. Absence of letters reflects an absence of significant differences.

There was strong evidence of lower nocturnal activity in solar fields than in the two controls for all species. The same pattern was found for their nocturnal occurrence, except for *P. pipistrellus* and *Plecotus spp.,* where no evidence was found of a difference between plot types (Fig 4, S5 Table in S1 File).

**Fig 5:**
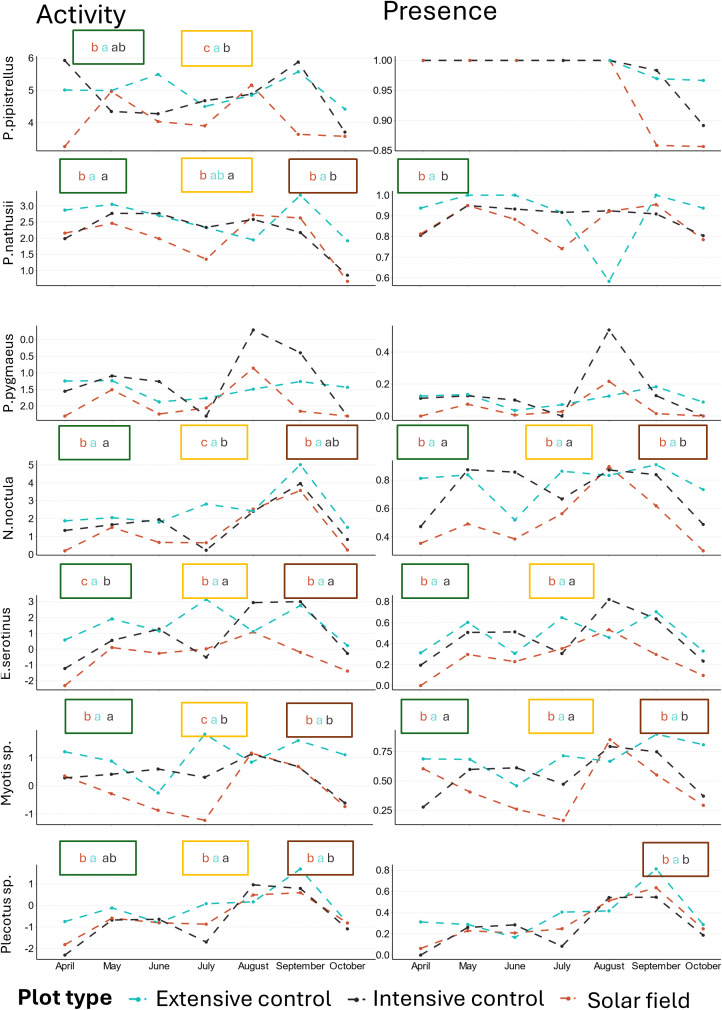
Mean of activity (left) and of occurrence (right) per month of each species in the three plot types. The three plot types are solar fields (in red), extensive controls (in blue), and intensive controls (in black). Significant differences between plot types per season (Spring (April-June) = green, Summer (June to September)= yellow, Autumn (September to October)= brown) are indicated by different letters (p < 0.05), with letter order representing the direction of the effect **(a > b)**. No letter indicates no significant difference. Activity (y-axis of the left panels) is log-transformed.

The two months that were the most sampled were May and June. Solar fields showed a consistently lower activity and occurrence than the two controls at all temporal scales measured than the two controls (Fig 5, S6 Table in S1 File).

## Discussion

This study builds upon the growing body of research exploring the impact of solar fields on biodiversity. Specifically, it examines how existing solar fields affect bat activity, species known for their sensitivity to land-use changes and widely recognised as bioindicators of ecosystem health [38]. We compared bat diversity, occurrence, and activity in solar fields with those in intensively managed grasslands and extensively managed meadows, habitats known to negatively and positively influence bat populations, respectively [2,20].

In this study, all bat species exhibited reduced presence and activity in solar fields compared to the controls (Figs 4 and 5). Even through part of results were based on automated classification, with 82 ± 12% of true positives, we assumed that the classification was reliable. Notably, even intensively managed grasslands demonstrated higher bat activity and presence than solar fields (Fig 4). Throughout the year, bat activity was the highest in extensive meadows and the lowest in solar fields, a pattern consistent across all species except for *P. pygmaeus* (Fig 5). These findings suggest that solar fields in our study are failing to provide potential useful habitat for all bat species.

Two species groups showed a weaker habitat avoidance of solar fields compared with the controls: *P. pipistrellus* and *Plecotus spp. P. pipistrellus* is a ubiquitous and highly synanthropic species, and it is the most common bat species in western Europe [39,40]. This characteristic might explain its presence in all three plot types on nearly every night surveyed. In contrast, *Plecotus spp.* are strongly associated with forested habitats such as forests, orchards, or parks [39]. The focus of this study on grasslands as an alternative habitat for solar fields may explain the limited use of these habitats by *Plecotus spp.* and consequently the absence of occurrence differences between plot types.

### Habitat connectivity is more important than habitat quantity

In this study, the community compositions of bats across the three plot types exhibited considerable homogeneity, as all bat species were present in the three plot types. The study locations were situated within a farmed landscape, characterised by its homogeneity and its scarce number of natural habitats.

These findings contrast with previous research in the literature. The amounts of forest and freshwater patches are well-established predictors of bat diversity and activity [41]. However, the specific effects of landscape composition and configuration parameters can be species-specific, complicating the selection of unique landscape metrics to explain the entire bat community composition (Fig 2). For instance, Fuentes-Montemayor et al. (2011) found that *P. pipistrellus* and *P. pygmaeus* exhibited different responses to landscape connectivity and the scales at which parameters were measured. Additionally, the seven bat species of the present study have varied ecologies, such as *N. noctula*, which can travel up to 26 km from their roosts, and *Plecotus* species, which typically remain within five kilometres of their roost. These examples underscore the species-specific nature of landscape effects and the challenges in selecting universal landscape metrics to explain bat community composition. Nevertheless, this study emphasises the need for connectivity between forested and freshwater patches in the landscape.

### Potential negative impact of solar fields

The present study demonstrates a clear reduction in the occurrence and activity of bats within solar fields in the agricultural landscape of the Netherlands compared with surrounding grasslands. The findings suggest that solar fields may not contribute to bat conservation, but rather reduce the habitat quality of bats in farmlands. To confirm this, comparing bat activity and occurrences before and after solar field construction would be needed, as all the studies known to the authors are spatial comparisons. Our results are consistent with a previous study comparing the edges of solar fields with the edges of adjacent control sites in the UK [16]. Unlike for *Plecotus spp.*, Tinsley et al. (2023) also found a reduction in activity at the edges of solar fields compared to the edges of controls for the same bat species as in this study. Potential reasons for the reduction in bat activity cited included a lack of prey (insects) and the collision risk posed by the clutter of solar panels [42].

The hypothesis concerning the lack of prey requires further exploration. Barré et al. (2024) found that bats have a straighter and faster flight behaviour in solar fields associated with a reduction of feeding behaviour. A study measuring insect abundance in the same solar fields as the present study found that solar fields had fewer ground-emerging Diptera and Coleoptera compared to control sites (Kocsis et al., forthcoming). These two orders are known to be important food resources for bats [43]. Therefore, a better comprehension of prey availability as well as the bat capabilities to detect and catch them within solar fields is essential to fully understand the impact of solar fields on bat activity. Therefore, to reduce the negative impact of solar fields on bats, it is necessary to enhance their suitability for flying insects while minimising the risk of bat collisions. Future exploration should also focus on providing direct observations and identification of the bat species. Indeed, direct recording might reduce the error produced by automated detection and would allow for bat behaviour recording at solar fields.

In the present study, bat activity and occurrence did not appear to be influenced by seasonality. Across both monthly and seasonal scales, solar fields were consistently less visited than the two grassland control sites. This absence of seasonal variation was unexpected, as bats are known to adjust their space use in response to seasonal changes and agricultural practices [22,44]. Recent work suggests that bats may use solar fields selectively depending on the season [45], which contrasts with our findings. One possible explanation for this discrepancy is variation in sampling effort across the annual cycle (S1 Fig). Both Szoldatits et al. (2025) and the present study focused on sampling between May and September, potentially overlooking periods of different space-use patterns. Future studies should therefore extend monitoring to early spring and late autumn, when bat activity and habitat use may differ and could reveal seasonal effects not captured here.

### Perspective of solar fields for bat conservation

This study demonstrates that current solar fields do not support the occurrence or activity of bat species. Although developers often commit to implementing mitigation or enhancement measures during construction, these are rarely applied effectively in practice [19]. Nevertheless, the negative impacts of solar fields on bats could be reduced through targeted ecological enhancement and by prioritising installations on intensively used farmland. For instance, integrating features such as hedgerows, tree lines, and flower-rich grasslands into solar developments could improve landscape connectivity and boost local insect abundance and diversity [20,21]. In the present study, bat detectors were placed at the edges of solar fields, and our results suggest that these edges did not enhance local habitat quality for bats. However, the six solar fields differed in edge characteristics, including either waterways, simple fences, or hedgerows (S1 Table in S1 File), and the hedgerows themselves varied in their stage of development. The small sample size prevented a comparison of bat activity and occurrence among edge types. Nevertheless, solar field edges have strong potential to enhance habitat quality within intensive agricultural landscapes. Indeed, well-developed hedgerows with a woody structure and mature trees are known to increase bat activity. Although trees can shade solar panels, this constraint could be addressed through differentiated edge management, such as establishing tall, woody hedgerows on the northern side of solar fields [46,47]. Future studies should explicitly test how solar field edge type and management influence bat activity and occurrence to determine whether solar fields can contribute positively to landscape quality for bat species. Solar fields have the potential to address both the energy and biodiversity crises, but at present, they fail to support bat communities, even within the already degraded agricultural environments.

## Supporting information

S1 File This file contains all supporting information tables (S1 to S6 Tables). **S1 Table.** Solar fields included in the survey; **S2 Table**. Manual annotation of one percent of each species automatically classified by kaleidoscope**; S3 Table**. Results of the generalised linear mixed models (GLMM) with a negative binomial distribution assessing the effect of; **S4 Table:** Results of the generalised linear mixed models (GLMM) with a Gaussian distribution assessing the effect of plot types on species diversity; **S5 Table.** Results of the generalised linear mixed model (GLMM) with a binomial or negative binomial (nbinom) distribution assessing the effect of plot types on species presence or activity, respectively; **S6 Table:** Results of the generalised linear mixed model (GLMM) with a binomial or negative binomial (nbinom) distribution assessing the effect of plot types on species presence or activity respectively. The model were performed for each season and species separately.
(DOCX)

S1 Fig Number of day and month sampled (Effort) in each solar fields varied due to field work constrained. (TIF)

S2 Fig Position of a bat detector on a solar panel structure. The microphone was directed away from the panel (On the left of the picture).
(TIF)
