# Supplementary material for: Solar fields in farmlands, their impact on bat presence and activity
Source: PLoS One. 2026 Jun 1;21(6):e0335581. doi: 10.1371/journal.pone.0335581 (PMC13225426; doi:10.1371/journal.pone.0335581)
Supplement: S1 File — S1 Table. Solar fields included in the survey; S2 Table. Manual annotation of one percent of each species automatically classified by kaleidoscope; S3 Table. Results of the generalised linear mixed models (GLMM) with a negative binomial distribution assessing the effect of; S4 Table: Results of the generalised linear mixed models (GLMM) with a Gaussian distribution assessing the effect of plot types on species diversity; S5 Table. Results of the generalised linear mixed model (GLMM) with a binomial or negative binomial (nbinom) distribution assessing the effect of plot types on species presence or activity, respectively; S6 Table: Results of the generalised linear mixed model (GLMM) with a binomial or negative binomial (nbinom) distribution assessing the effect of plot types on species presence or activity respectively. The model were performed for each season and species separately. (DOCX) [file pone.0335581.s001.docx]

**Supporting Information 1:** Solar fields included in the survey. Information was communicated by the developers. Grazing: extensive (ext) with duration in brackets or intensive pressure grazing (ipg) with times done per year in brackets. Panel orientations are south-facing (S) or East-West-facing (EW).

| **Code** | **Region** | **Construction year** | **Previous land-use** | **Size (ha)** | **Ground coverage (%)** | **Mowing (per year)** | **Mowing waterway (per year)** | **Grazing** | **Sown** | **Panels orientation** | **Edge** | **Panel to Edge (cm)** |
| --- | --- | --- | --- | --- | --- | --- | --- | --- | --- | --- | --- | --- |
| **1** | North Brabant | 2021 | arable farming | 33.2 | NA | 0 | Non existing | ipg (3) | yes | EW | Hedgerow | 404 |
| **2** | Zeeland | 2020 | NA | 33 | NA | 2 | 1 side every 2 years | no | yes | S | Waterway | 545 |
| **3** | Zeeland | 2021 | arable farming | 17 | 61 | 2 | 1 | no | yes | S | Hedgerow | 81 |
| **4** | Utrecht | 2021 | grassland | 16.5 | 68 | 5 | 1 | no | no | S | Fence | 92 |
| **5** | Friesland | 2018 | field | 16.4 | 65 | 2 | non-existing | no | no | S | Hedgerow | 1079 |
| **6** | Groningen | 2018 | grassland | 8 | 73 | 3 | Non-existing | ext | no | s | Hedgerow | NA |

**Supporting Information 2:** Manual annotation of on one percent of each species automatically classified by kaleidoscope (First column). Verification was done by one observer.

| Kaleidoscope | E. serotinus | N. noctula | P. pipistrellus | Myotis sp. | P. nathusii | P. pygmaeus | Plecotus sp. | Unknown + Noise | Sample | True positive |
| --- | --- | --- | --- | --- | --- | --- | --- | --- | --- | --- |
| E. serotinus | 46 | 4 | 4 | 0 | 0 | 0 | 0 | 2 | 56 | 0.82 |
| Myotis sp. | 0 | 0 | 0 | 63 | 17 | 0 | 0 | 3 | 83 | 0.76 |
| N. noctula | 0 | 146 | 2 | 0 | 1 | 0 | 0 | 5 | 154 | 0.95 |
| P. nathusii | 1 | 2 | 23 | 1 | 227 | 0 | 0 | 3 | 257 | 0.88 |
| P. pipistrellus | 2 | 2 | 1017 | 0 | 5 | 0 | 0 | 4 | 1030 | 0.99 |
| P. pygmaeus | 1 | 0 | 6 | 0 | 2 | 30 | 0 | 9 | 48 | 0.63 |
| Plecotus sp. | 0 | 1 | 0 | 0 | 0 | 0 | 8 | 1 | 10 | 0.80 |

**Supporting Information 3:** Results of the generalised linear mixed models (GLMM) with a negative binomial distribution assessing the effect of forest patches and freshwater patches connectivity on each species' night activity. Location (n=6) was incorporated as a random variable. The Week number was also added as a covariate to correct temporal variation when it improved the model. For *N. noctula* the effort (number of days the bat detector was recording) was also included. The estimates and standard errors (SE) of the intercept and for the landscape variables (ext = Extensive control and int = Intensive control) are reported for every model.

| **Species** | **Covariates** | **Estimate** | **SE** | **z value** |
| --- | --- | --- | --- | --- |
| Plecotus sp. | (Intercept) | -11.97 | 2.73 | -4.39 |
| Plecotus sp. | Forest (2km) | 0.04 | 0.04 | 0.93 |
| Plecotus sp. | Freshwater (500m) | 0.30 | 0.14 | 2.10 |
| Plecotus sp. | Week | 0.03 | 0.01 | 4.81 |
| Myotis sp. | (Intercept) | -1.06 | 1.29 | -0.82 |
| Myotis sp. | Forest (2km) | 0.03 | 0.02 | 1.45 |
| Myotis sp. | Freshwater (500m) | 0.04 | 0.07 | 0.56 |
| E.serotinus | (Intercept) | -3.11 | 2.29 | -1.36 |
| E.serotinus | Forest (2km) | -0.02 | 0.04 | -0.57 |
| E.serotinus | Freshwater (500m) | 0.28 | 0.12 | 2.34 |
| N.noctula | (Intercept) | -3.90 | 2.62 | -1.49 |
| N.noctula | Forest (2km) | 0.03 | 0.04 | 0.78 |
| N.noctula | Freshwater (500m) | 0.30 | 0.13 | 2.21 |
| N.noctula | Effort | 0.00 | 0.01 | 0.11 |
| P.pygmaeus | (Intercept) | -2.10 | 1.52 | -1.38 |
| P.pygmaeus | Forest (2km) | -0.01 | 0.02 | -0.41 |
| P.pygmaeus | Freshwater (500m) | 0.03 | 0.08 | 0.40 |
| P.pygmaeus | Week | -0.01 | 0.01 | -0.63 |
| P.nathusii | (Intercept) | 1.98 | 0.96 | 2.05 |
| P.nathusii | Forest (2km) | 0.04 | 0.01 | 2.38 |
| P.nathusii | Freshwater (500m) | 0.02 | 0.05 | 0.31 |
| P.nathusii | Week | -0.03 | 0.00 | -7.34 |
| P.pipistrellus | (Intercept) | 1.72 | 1.16 | 1.48 |
| P.pipistrellus | Forest (2km) | 0.04 | 0.02 | 2.39 |
| P.pipistrellus | Freshwater (500m) | 0.13 | 0.06 | 2.21 |
| P.pipistrellus | Week | -0.05 | 0.00 | -10.00 |

**Supporting Information 4:** Results of the generalised linear mixed models (GLMM) with a Gaussian distribution assessing the effect of plot types on species diversity. Location (n=6) was incorporated as a random variable. The estimates and standard errors (SE) of the intercept (Solar) and for the two controls (ext = Extensive control and int = Intensive control) are reported for every model. The sum of the nights sampled was 518 nights for the solar fields, 398 nights for the extensive controls and 402 nights for the intensive controls.

| Hill number | Field type | Estimate | SE | Z-Value |
| --- | --- | --- | --- | --- |
| Shannon | Solar (Intercept) | 4.81 | 0.34 | 14.18 |
|  | Ext | 0.98 | 0.31 | 3.17 |
|  | Int | 1.03 | 0.33 | 3.11 |
| Simpson | Solar (Intercept) | 4.30 | 0.35 | 12.23 |
|  | Ext | 1.09 | 0.35 | 3.13 |
|  | Int | 1.14 | 0.38 | 3.03 |
| Species Richness | Solar (Intercept) | 6.50 | 0.19 | 35.09 |
|  | Ext | 0.50 | 0.26 | 1.91 |
|  | Int | 0.50 | 0.29 | 1.72 |

**Supporting information 5:** Results of the generalised linear mixed model (GLMM) with a binomial or negative binomial (nbinom) distribution assessing the effect of plot types on species presence or activity, respectively. The models were performed for each species separately. Location (n=6) was incorporated as a random variable. The estimates and standard errors (SE) of the intercept (Solar) and for the two controls (extensive = Extensive control and intensive = Intensive control) are reported for every model. The Julian day or the yearly effort (number of nights sampled) was incorporated as a covariate when it improved the Akaike Information criterion by more than 2. The sum of the nights sampled was 518 nights for the solar fields, 398 nights for the extensive controls and 402 nights for the intensive controls.

| **Response Variables** | **Species** | **Covariates** | **Estimate** | **SE** | **z value** |
| --- | --- | --- | --- | --- | --- |
| Presence | Plecotus sp. | Solar (Intercept) | -5.64 | 0.54 | -10.48 |
| Presence | Plecotus sp. | Extensive | 0.20 | 0.15 | 1.30 |
| Presence | Plecotus sp. | Intensive | 0.08 | 0.15 | 0.54 |
| Activity | Plecotus sp. | Solar (Intercept) | -5.69 | 0.58 | -9.76 |
| Activity | Plecotus sp. | Extensive | 0.56 | 0.14 | 4.07 |
| Activity | Plecotus sp. | Intensive | 0.60 | 0.14 | 4.31 |
| Activity | Myotis sp. | Solar (Intercept) | -1.06 | 1.29 | -0.82 |
| Presence | Myotis sp. | Solar (Intercept) | -0.31 | 0.23 | -1.37 |
| Presence | Myotis sp. | Extensive | 1.16 | 0.14 | 8.04 |
| Presence | Myotis sp. | Intensive | 0.80 | 0.14 | 5.68 |
| Activity | Myotis sp. | Solar (Intercept) | -0.20 | 0.19 | -1.03 |
| Activity | Myotis sp. | Extensive | 1.13 | 0.10 | 10.89 |
| Activity | Myotis sp. | Intensive | 0.80 | 0.11 | 7.26 |
| Presence | E.serotinus | Solar (Intercept) | -0.96 | 0.19 | -5.10 |
| Presence | E.serotinus | Extensive | 0.94 | 0.14 | 6.58 |
| Presence | E.serotinus | Intensive | 0.97 | 0.14 | 6.72 |
| Activity | E.serotinus | Solar (Intercept) | -0.16 | 0.33 | -0.50 |
| Activity | E.serotinus | Extensive | 1.69 | 0.19 | 9.01 |
| Activity | E.serotinus | Intensive | 1.26 | 0.16 | 7.89 |
| Presence | N.noctula | Solar (Intercept) | -4.29 | 0.33 | -13.20 |
| Presence | N.noctula | Extensive | 1.41 | 0.16 | 9.08 |
| Presence | N.noctula | Intensive | 1.21 | 0.15 | 7.85 |
| Activity | N.noctula | Solar (Intercept) | 48.00 | 21.15 | 2.27 |
| Activity | N.noctula | Extensive | 1.30 | 0.13 | 10.18 |
| Activity | N.noctula | Intensive | 0.73 | 0.13 | 5.77 |
| Activity | N.noctula | Julian | 0.00 | 0.00 | -2.22 |
| Presence | P.pygmaeus | Solar (Intercept) | -7.61 | 0.29 | -26.15 |
| Presence | P.pygmaeus | Extensive | 1.23 | 0.26 | 4.76 |
| Presence | P.pygmaeus | Intensive | 1.12 | 0.26 | 4.29 |
| Activity | P.pygmaeus | Solar (Intercept) | -3.47 | 0.74 | -4.69 |
| Activity | P.pygmaeus | Extensive | 0.88 | 0.32 | 2.70 |
| Activity | P.pygmaeus | Intensive | 1.17 | 0.30 | 3.93 |
| Activity | P.pygmaeus | Yearly Effort | 0.01 | 0.01 | 1.01 |
| Presence | P.nathusii | Solar (Intercept) | 2.93 | 0.58 | 5.04 |
| Presence | P.nathusii | Extensive | 0.65 | 0.25 | 2.65 |
| Presence | P.nathusii | Intensive | 0.07 | 0.21 | 0.34 |
| Activity | P.nathusii | Solar (Intercept) | 91.92 | 12.05 | 7.63 |
| Activity | P.nathusii | Extensive | 0.49 | 0.07 | 6.64 |
| Activity | P.nathusii | Intensive | 0.33 | 0.08 | 4.24 |
| Activity | P.nathusii | Julian | 0.00 | 0.00 | -7.44 |
| Presence | P.pipistrellus | Solar (Intercept) | 1.18 | 1.66 | 0.71 |
| Presence | P.pipistrellus | Extensive | 0.30 | 0.40 | 0.76 |
| Presence | P.pipistrellus | Intensive | -0.18 | 0.41 | -0.44 |
| Activity | P.pipistrellus | Solar (Intercept) | 142.84 | 13.20 | 10.82 |
| Activity | P.pipistrellus | Extensive | 0.57 | 0.12 | 4.74 |
| Activity | P.pipistrellus | Intensive | 0.38 | 0.11 | 3.58 |
| Activity | P.pipistrellus | Julian | -0.01 | 0.00 | -10.62 |
| Activity | P.pipistrellus | Yearly Effort | 0.01 | 0.00 | 2.01 |

**Supporting information 6:** Results of the generalised linear mixed model (GLMM) with a binomial or negative binomial (nbinom) distribution assessing the effect of plot types on species presence or activity respectively. The model were performed for each season and species separately. Location (n=6) was incorporated as a random variable. The estimates and standard errors (SE) of the intercept (Solar) and for the two controls (extensive = Extensive control and intensive = Intensive control) are reported for every model. The sum of the nights sampled was 238 nights for the solar fields, 194 nights for the extensive controls and 226 nights for the intensive controls in spring, 162 nights for the solar fields, 113 nights for the extensive controls and 75 nights for the intensive controls in summer, and 118 nights for the solar fields, 101 nights for the extensive controls and 91 nights for the intensive controls in autumn.

| **Response Variable** | **Species** | **Season** | **Plot** | **Est.** | **SE** | **z value** |
| --- | --- | --- | --- | --- | --- | --- |
| presence | Plecotus sp. | Autumn | Solar field (Intercept) | -4.55 | 0.28 | -15.97 |
| presence | Plecotus sp. | Autumn | Extensive | -0.13 | 0.30 | -0.43 |
| presence | Plecotus sp. | Autumn | Intensive | -0.86 | 0.34 | -2.52 |
| presence | Plecotus sp. | Summer | Solar field (Intercept) | -3.97 | 0.86 | -4.63 |
| presence | Plecotus sp. | Summer | Extensive | 0.16 | 0.34 | 0.47 |
| presence | Plecotus sp. | Summer | Intensive | 0.28 | 0.29 | 0.95 |
| presence | Plecotus sp. | Spring | Solar field (Intercept) | -5.41 | 0.45 | -12.06 |
| presence | Plecotus sp. | Spring | Extensive | 0.52 | 0.23 | 2.27 |
| presence | Plecotus sp. | Spring | Intensive | 0.53 | 0.24 | 2.23 |
| activity | Plecotus sp. | Autumn | Solar field (Intercept) | -3.78 | 2.07 | -1.82 |
| activity | Plecotus sp. | Autumn | Extensive | -0.16 | 0.26 | -0.63 |
| activity | Plecotus sp. | Autumn | Intensive | -0.71 | 0.30 | -2.33 |
| activity | Plecotus sp. | Summer | Solar field (Intercept) | -4.48 | 0.74 | -6.05 |
| activity | Plecotus sp. | Summer | Extensive | 1.07 | 0.25 | 4.36 |
| activity | Plecotus sp. | Summer | Intensive | 1.20 | 0.20 | 6.05 |
| activity | Plecotus sp. | Spring | Solar field (Intercept) | -5.31 | 0.47 | -11.21 |
| activity | Plecotus sp. | Spring | Extensive | 0.68 | 0.20 | 3.35 |
| activity | Plecotus sp. | Spring | Intensive | 0.39 | 0.22 | 1.75 |
| presence | Myotis sp. | Autumn | Solar field (Intercept) | -4.61 | 0.20 | -22.94 |
| presence | Myotis sp. | Autumn | Extensive | 1.58 | 0.30 | 5.29 |
| presence | Myotis sp. | Autumn | Intensive | 0.32 | 0.29 | 1.08 |
| presence | Myotis sp. | Summer | Solar field (Intercept) | -3.29 | 0.33 | -10.09 |
| presence | Myotis sp. | Summer | Extensive | 1.62 | 0.34 | 4.75 |
| presence | Myotis sp. | Summer | Intensive | 1.46 | 0.28 | 5.16 |
| presence | Myotis sp. | Spring | Solar field (Intercept) | -4.19 | 0.29 | -14.54 |
| presence | Myotis sp. | Spring | Extensive | 1.21 | 0.20 | 5.92 |
| presence | Myotis sp. | Spring | Intensive | 1.05 | 0.21 | 4.98 |
| activity | Myotis sp. | Autumn | Solar field (Intercept) | -3.42 | 1.12 | -3.05 |
| activity | Myotis sp. | Autumn | Extensive | 1.48 | 0.21 | 6.99 |
| activity | Myotis sp. | Autumn | Intensive | 0.08 | 0.25 | 0.30 |
| activity | Myotis sp. | Summer | Solar field (Intercept) | -3.21 | 0.27 | -12.06 |
| activity | Myotis sp. | Summer | Extensive | 1.79 | 0.20 | 8.99 |
| activity | Myotis sp. | Summer | Intensive | 1.17 | 0.18 | 6.47 |
| activity | Myotis sp. | Spring | Solar field (Intercept) | -4.33 | 0.27 | -15.85 |
| activity | Myotis sp. | Spring | Extensive | 1.31 | 0.13 | 9.92 |
| activity | Myotis sp. | Spring | Intensive | 1.15 | 0.14 | 8.15 |
| presence | E.serotinus | Autumn | Solar field (Intercept) | -4.76 | 1.85 | -2.57 |
| presence | E.serotinus | Autumn | Extensive | 0.78 | 0.35 | 2.22 |
| presence | E.serotinus | Autumn | Intensive | 0.76 | 0.36 | 2.10 |
| presence | E.serotinus | Summer | Solar field (Intercept) | -3.63 | 0.38 | -9.64 |
| presence | E.serotinus | Summer | Extensive | 1.77 | 0.33 | 5.27 |
| presence | E.serotinus | Summer | Intensive | 1.54 | 0.29 | 5.27 |
| presence | E.serotinus | Spring | Solar field (Intercept) | -5.00 | 0.24 | -21.24 |
| presence | E.serotinus | Spring | Extensive | 1.49 | 0.21 | 7.07 |
| presence | E.serotinus | Spring | Intensive | 1.33 | 0.22 | 6.04 |
| activity | E.serotinus | Autumn | Solar field (Intercept) | -4.49 | 1.60 | -2.80 |
| activity | E.serotinus | Autumn | Extensive | 1.23 | 0.40 | 3.04 |
| activity | E.serotinus | Autumn | Intensive | 1.04 | 0.39 | 2.64 |
| activity | E.serotinus | Summer | Solar field (Intercept) | -3.47 | 0.57 | -6.07 |
| activity | E.serotinus | Summer | Extensive | 2.89 | 0.30 | 9.48 |
| activity | E.serotinus | Summer | Intensive | 2.34 | 0.24 | 9.93 |
| activity | E.serotinus | Spring | Solar field (Intercept) | -4.26 | 0.31 | -13.73 |
| activity | E.serotinus | Spring | Extensive | 2.18 | 0.23 | 9.36 |
| activity | E.serotinus | Spring | Intensive | 0.89 | 0.23 | 3.94 |
| presence | N.noctula | Autumn | Solar field (Intercept) | -3.91 | 1.54 | -2.53 |
| presence | N.noctula | Autumn | Extensive | 0.96 | 0.30 | 3.19 |
| presence | N.noctula | Autumn | Intensive | 0.15 | 0.30 | 0.49 |
| presence | N.noctula | Summer | Solar field (Intercept) | -2.24 | 0.43 | -5.24 |
| presence | N.noctula | Summer | Extensive | 1.62 | 0.45 | 3.58 |
| presence | N.noctula | Summer | Intensive | 1.07 | 0.35 | 3.08 |
| presence | N.noctula | Spring | Solar field (Intercept) | -3.89 | 0.37 | -10.50 |
| presence | N.noctula | Spring | Extensive | 1.72 | 0.23 | 7.46 |
| presence | N.noctula | Spring | Intensive | 2.20 | 0.26 | 8.38 |
| activity | N.noctula | Autumn | Solar field (Intercept) | -2.73 | 0.20 | -13.42 |
| activity | N.noctula | Autumn | Extensive | 0.98 | 0.26 | 3.83 |
| activity | N.noctula | Autumn | Intensive | 0.56 | 0.27 | 2.13 |
| activity | N.noctula | Autumn | LocationMA | -1.74 | 0.21 | -8.15 |
| activity | N.noctula | Autumn | LocationOO | 7.04 | 1.18 | 5.98 |
| activity | N.noctula | Autumn | LocationTH | -19.10 | 3666.73 | -0.01 |
| activity | N.noctula | Summer | Solar field (Intercept) | -1.42 | 0.17 | -8.34 |
| activity | N.noctula | Summer | Extensive | 2.41 | 0.21 | 11.66 |
| activity | N.noctula | Summer | Intensive | 1.14 | 0.18 | 6.47 |
| activity | N.noctula | Summer | LocationAD | -1.96 | 0.28 | -7.03 |
| activity | N.noctula | Summer | LocationMA | -1.13 | 0.21 | -5.29 |
| activity | N.noctula | Summer | LocationOO | 2.36 | 0.25 | 9.50 |
| activity | N.noctula | Summer | LocationRO | -0.72 | 0.26 | -2.74 |
| activity | N.noctula | Summer | LocationTH | -1.08 | 0.42 | -2.57 |
| activity | N.noctula | Spring | Solar field (Intercept) | -1.95 | 0.12 | -15.88 |
| activity | N.noctula | Spring | Extensive | 1.28 | 0.15 | 8.76 |
| activity | N.noctula | Spring | Intensive | 1.26 | 0.15 | 8.39 |
| activity | N.noctula | Spring | LocationAD | -2.47 | 0.21 | -11.83 |
| activity | N.noctula | Spring | LocationMA | -2.01 | 0.18 | -11.15 |
| activity | N.noctula | Spring | LocationOO | -0.78 | 0.18 | -4.37 |
| activity | N.noctula | Spring | LocationRO | -1.58 | 0.18 | -8.61 |
| activity | N.noctula | Spring | LocationTH | -0.33 | 0.25 | -1.35 |
| presence | P.pygmaeus | Autumn | Solar field (Intercept) | -36.18 | 7282733.31 | 0.00 |
| presence | P.pygmaeus | Autumn | Extensive | 34.48 | 7282733.31 | 0.00 |
| presence | P.pygmaeus | Autumn | Intensive | 32.61 | 7282733.31 | 0.00 |
| presence | P.pygmaeus | Summer | Solar field (Intercept) | -2.36 | 0.91 | -2.59 |
| presence | P.pygmaeus | Summer | Extensive | 1.12 | 1.32 | 0.85 |
| presence | P.pygmaeus | Summer | Intensive | 0.90 | 1.48 | 0.61 |
| presence | P.pygmaeus | Spring | Solar field (Intercept) | 8.23 | 6.96 | 1.18 |
| presence | P.pygmaeus | Spring | Extensive | 14.67 | 11.14 | 1.32 |
| presence | P.pygmaeus | Spring | Intensive | 39.27 | 1612028.59 | 0.00 |
| activity | P.pygmaeus | Autumn | Solar field (Intercept) | -25.15 | NA | NA |
| activity | P.pygmaeus | Autumn | Extensive | 23.00 | NA | NA |
| activity | P.pygmaeus | Autumn | Intensive | 23.72 | NA | NA |
| activity | P.pygmaeus | Summer | Solar field (Intercept) | -2.20 | 0.38 | -5.85 |
| activity | P.pygmaeus | Summer | Extensive | 0.12 | 0.47 | 0.26 |
| activity | P.pygmaeus | Summer | Intensive | 0.89 | 0.38 | 2.33 |
| activity | P.pygmaeus | Spring | Solar field (Intercept) | -3.03 | 0.59 | -5.17 |
| activity | P.pygmaeus | Spring | Extensive | 0.80 | 0.54 | 1.49 |
| activity | P.pygmaeus | Spring | Intensive | 1.10 | 0.53 | 2.07 |
| presence | P.nathusii | Autumn | Solar field (Intercept) | 1.47 | 0.92 | 1.61 |
| presence | P.nathusii | Autumn | Extensive | 0.60 | 0.32 | 1.87 |
| presence | P.nathusii | Autumn | Intensive | 0.05 | 0.31 | 0.15 |
| presence | P.nathusii | Summer | Solar field (Intercept) | 3.28 | 0.72 | 4.56 |
| presence | P.nathusii | Summer | Extensive | -0.54 | 0.67 | -0.81 |
| presence | P.nathusii | Summer | Intensive | 0.94 | 0.58 | 1.63 |
| presence | P.nathusii | Spring | Solar field (Intercept) | 3.13 | 0.60 | 5.21 |
| presence | P.nathusii | Spring | Extensive | 2.80 | 1.04 | 2.68 |
| presence | P.nathusii | Spring | Intensive | -0.27 | 0.41 | -0.67 |
| activity | P.nathusii | Autumn | Solar field (Intercept) | 0.97 | 0.32 | 3.04 |
| activity | P.nathusii | Autumn | Extensive | 1.16 | 0.19 | 5.98 |
| activity | P.nathusii | Autumn | Intensive | 0.01 | 0.20 | 0.05 |
| activity | P.nathusii | Summer | Solar field (Intercept) | 2.29 | 0.24 | 9.67 |
| activity | P.nathusii | Summer | Extensive | 0.32 | 0.15 | 2.06 |
| activity | P.nathusii | Summer | Intensive | 0.49 | 0.14 | 3.59 |
| activity | P.nathusii | Spring | Solar field (Intercept) | 2.31 | 0.20 | 11.63 |
| activity | P.nathusii | Spring | Extensive | 0.52 | 0.09 | 5.91 |
| activity | P.nathusii | Spring | Intensive | 0.33 | 0.10 | 3.43 |
| presence | P.pipistrellus | Autumn | Solar field (Intercept) | -0.80 | 0.91 | -0.89 |
| presence | P.pipistrellus | Autumn | Extensive | -0.46 | 0.51 | -0.91 |
| presence | P.pipistrellus | Autumn | Intensive | -0.66 | 0.50 | -1.34 |
| presence | P.pipistrellus | Summer | Solar field (Intercept) | 6.35 | 4.71 | 1.35 |
| presence | P.pipistrellus | Summer | Extensive | 29.66 | 1189717.69 | 0.00 |
| presence | P.pipistrellus | Summer | Intensive | 19.13 | 34661.83 | 0.00 |
| presence | P.pipistrellus | Spring | Solar field (Intercept) | 30.48 | 1693000.84 | 0.00 |
| presence | P.pipistrellus | Spring | Extensive | 5.90 | 32343647.92 | 0.00 |
| presence | P.pipistrellus | Spring | Intensive | 5.67 | 31551940.54 | 0.00 |
| activity | P.pipistrellus | Autumn | Solar field (Intercept) | -1.14 | 1.14 | -0.99 |
| activity | P.pipistrellus | Autumn | Extensive | 0.54 | 0.28 | 1.89 |
| activity | P.pipistrellus | Autumn | Intensive | 0.01 | 0.29 | 0.05 |
| activity | P.pipistrellus | Summer | Solar field (Intercept) | 0.25 | 0.27 | 0.94 |
| activity | P.pipistrellus | Summer | Extensive | 1.25 | 0.17 | 7.19 |
| activity | P.pipistrellus | Summer | Intensive | 0.81 | 0.15 | 5.49 |
| activity | P.pipistrellus | Spring | Solar field (Intercept) | -0.25 | 0.22 | -1.12 |
| activity | P.pipistrellus | Spring | Extensive | 0.52 | 0.12 | 4.38 |
| activity | P.pipistrellus | Spring | Intensive | 0.37 | 0.12 | 3.00 |
