# Supplementary figures and images for: Solar fields in farmlands, their impact on bat presence and activity
Source: PLoS One. 2026 Jun 1;21(6):e0335581. doi: 10.1371/journal.pone.0335581 (PMC13225426; doi:10.1371/journal.pone.0335581)

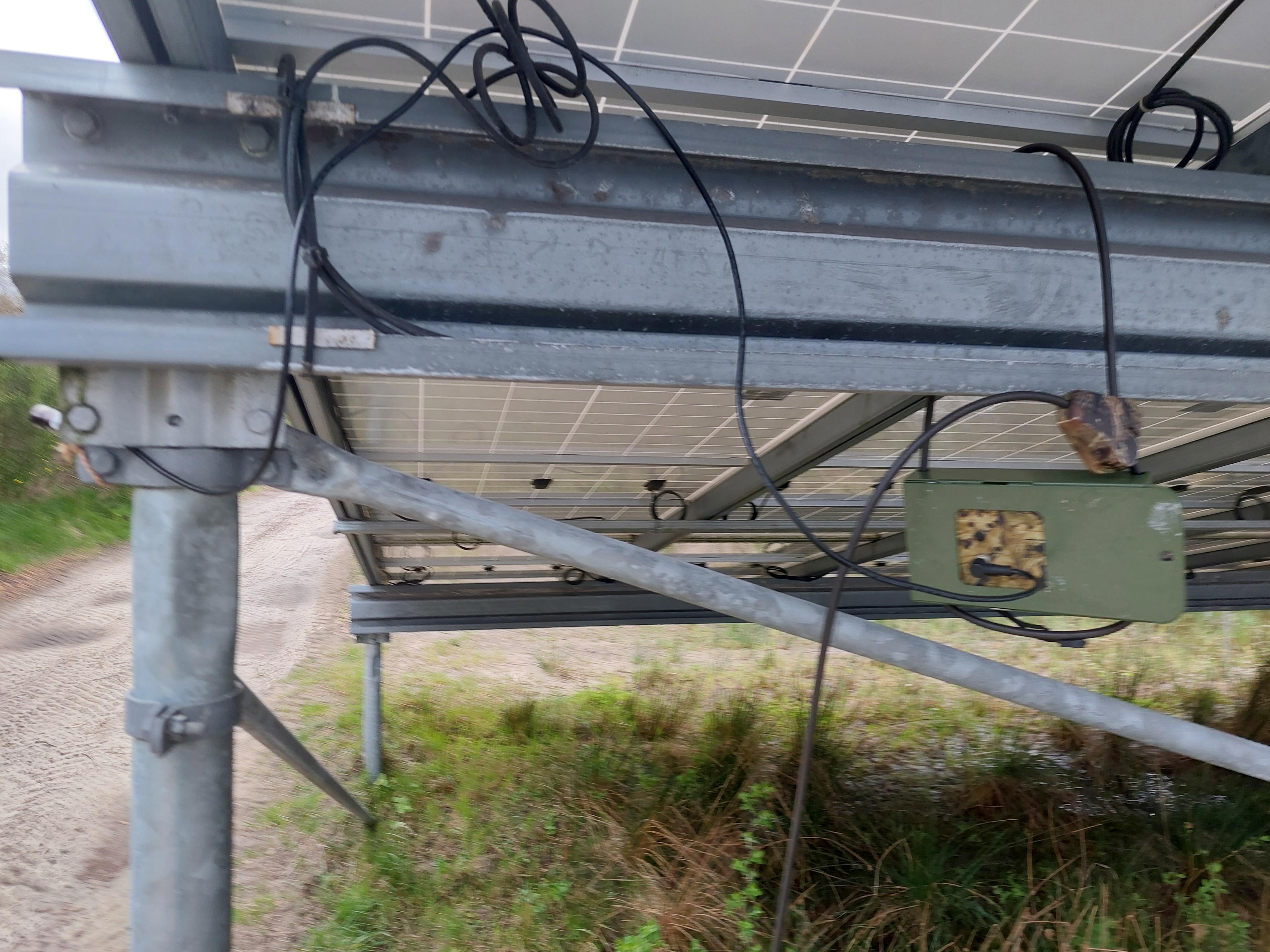

Supplement: S2 Fig — The microphone was directed away from the panel (On the left of the picture). (TIF) [file pone.0335581.s003.tif]
